# Protein‐Specific, Multicolor and 3D STED Imaging in Cells with DNA‐Labeled Antibodies

**DOI:** 10.1002/anie.201910115

**Published:** 2019-11-07

**Authors:** Christoph Spahn, Florian Hurter, Mathilda Glaesmann, Christos Karathanasis, Marko Lampe, Mike Heilemann

**Affiliations:** ^1^ Institute of Physical and Theoretical Chemistry Goethe-University Frankfurt Max-von-Laue-Str. 7 60438 Frankfurt Germany; ^2^ Advanced Light Microscopy Facility European Molecular Biology Laboratory Meyerhofstr. 1 69117 Heidelberg Germany

**Keywords:** DNA-PAINT, fluorescence, fluorescent probes, multicolor imaging, STED microscopy

## Abstract

Photobleaching is a major challenge in fluorescence microscopy, in particular if high excitation light intensities are used. Signal‐to‐noise and spatial resolution may be compromised, which limits the amount of information that can be extracted from an image. Photobleaching can be bypassed by using exchangeable labels, which transiently bind to and dissociate from a target, thereby replenishing the destroyed labels with intact ones from a reservoir. Here, we demonstrate confocal and STED microscopy with short, fluorophore‐labeled oligonucleotides that transiently bind to complementary oligonucleotides attached to protein‐specific antibodies. The constant exchange of fluorophore labels in DNA‐based STED imaging bypasses photobleaching that occurs with covalent labels. We show that this concept is suitable for targeted, two‐color STED imaging of whole cells.

## Graphical abstract

**Short, DNA‐based fluorophore labels** that transiently and repeatedly bind to DNA‐labeled antibodies enable super‐resolution STED imaging. Compared to covalent labels, dynamic labels constantly exchange between target‐bound and free states, which bypasses photobleaching and subsequently increases image quality and information content.

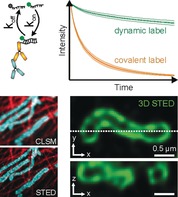

Super‐resolution stimulated emission depletion (STED) microscopy has contributed to our to‐date understanding of cell biology.^1^, ^2^ As with other fluorescence microscopy techniques that use comparably high laser intensities, photobleaching of the fluorophore labels limits image quality and information content. Various solutions to minimize photobleaching in STED microscopy have been introduced, including dynamic tuning of the excitation light during image acquisition,^3^ the development of photostable fluorophores,^4^ or the use of fluorophores with multiple off‐states.^5^ An alternative route is using fluorophore labels that reversibly bind to a target structure and exchange with a reservoir,^6^, ^7^ making STED microscopy insensitive to photobleaching and enabling multicolor and 3D imaging of whole cells.^8^ This is achieved by a permanent exchange of labels, which removes photobleached fluorophores and replenishes them with intact ones that are present in the imaging buffer. The benefits of this approach are i) STED imaging with high contrast, ii) multicolor imaging without special demands to protect spectrally distinct fluorophore species, iii) whole‐cell 3D imaging and large volume imaging, and iv) live‐cell imaging with long acquisition times.^8^ So far, this concept has been limited to a small number of labels, and not capable of targeting specific proteins in a cell.

Here, we introduce a target‐specific approach for STED microscopy with exchangeable fluorophore labels for the purpose of cell imaging. We exploit the transient and reversible binding of short, fluorophore‐labeled oligonucleotides (imager strand) to an antibody carrying a complementary oligonucleotide (docking strand), a concept used in DNA point accumulation for imaging in nanoscale topography (DNA‐PAINT).^9^ DNA‐PAINT is a single‐molecule localization microscopy technique^10^ and requires low nanomolar concentrations in order to separate binding events by a distance sufficiently large for single‐molecule detection. In order to be suitable for STED microscopy, a high labeling density is required to saturate all target binding sites.^11^ Higher concentrations of exchangeable fluorophore labels can achieve such a pseudo‐permanent labeling and enable STED imaging with minimized photobleaching.^8^, ^12^ DNA‐PAINT labels have been previously combined with STED microscopy using longer oligonucleotides for stable hybridization and denaturing washing buffers to exchange the labels between imaging rounds.^11^

We first explored the suitability of exchangeable DNA‐based fluorophore labels using confocal microscopy and immunofluorescence labeling, using a target‐specific primary antibody and a secondary antibody labeled with a docking strand (see the Methods section of the Supporting Information; Figure 1 A). In our previous work, we found that small‐molecule labels with a dissociation constant in the low micromolar range and a *k*_off_ of 1–50 s^−1^ ensure quasi‐continuous labeling.^8^ In order to tune the exchange of the label from the target, DNA oligonucleotides offer two parameters that can be tuned: the concentration of the imager strand in solution, which determines the on‐binding rate *k*_on_, and the length and sequence (GC content) of the hybridization pair, which determines the off‐binding rate *k*_off_. Profiting from available data in the field of single‐molecule DNA‐PAINT, we selected two previously characterized oligonucleotide sequences (termed P1 and P5, see the Methods section and Table S1 in the Supporting Information).^13^ In order to achieve a faster off‐binding (a larger off‐binding rate *k*_off_), we shortened the duplex length and used 8 and 9 nucleotides for P1 and 9 nucleotides for P5. We labeled the P1 imager strands with the fluorophore Abberior STAR 635P, which previously demonstrated excellent performance in STED microscopy,^14^ and the P5 imager strand with Alexa Fluor 594. We determined the binding times (1/*k*_off_) of the imager strands using single‐molecule DNA‐PAINT imaging, and found 209±3 ms (P1‐AbberiorSTAR635P, 8 nt duplex), 491±7 ms (P1‐AbberiorSTAR635P, 9 nt duplex), and 363±8 ms (P5‐AlexaFluor594, 9 nt duplex; Figure S1 and Note 1 in the Supporting Information). These values are in a similar range as those of other exchangeable fluorophore labels that have been successfully used for STED microscopy.^8^

**Figure 1:**
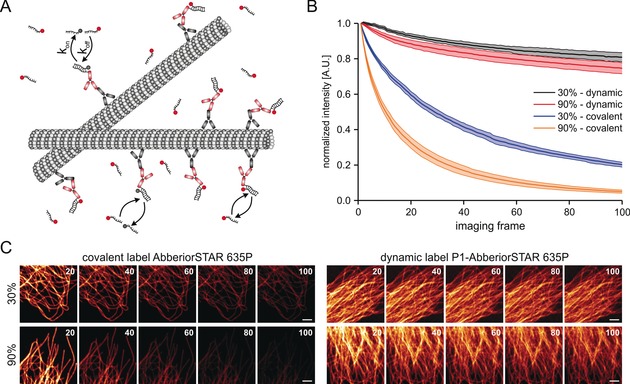
Pseudo‐permanent labeling of cellular structures with DNA‐based fluorophore labels. A) Labeling of cellular targets using secondary antibodies labeled with DNA docking strands and transiently binding fluorophore‐labeled oligonucleotides (imager strands). B) Intensity over time recorded on a confocal laser‐scanning microscope for the fluorophore Abberior STAR 635P either as a covalent label (fluorophore‐labeled secondary antibody) or as dynamic label (DNA‐labeled secondary antibody; P1‐AbberiorSTAR635P, 500 nm). C) Representative time series for covalent (left panel) and dynamic (right panel) labeling of microtubules at varying laser intensities. Shown are interval average images generated from 20 frames each (scale bars 3 μm).

We next performed a titration experiment to determine a suitable imager strand concentration for quasi‐continuous labeling of the microtubule cytoskeleton and extracted the signal‐to‐background and signal‐to‐noise ratio from confocal images (Supporting Information, Figure S2). We chose an imager strand concentration of 500 nm (P1‐AbberiorSTAR635P, 8 nt duplex) for further experiments because it has the highest signal‐to‐noise ratio and superior exchange kinetics (Supporting Information, Figures S1 and S2 D,E). Next, we compared the amount of photobleaching of Abberior STAR 635P conjugated to a secondary antibody (here termed covalent labeling) to DNA‐based labeling (dynamic labeling) (Figure 1 B and Supporting Information, Figure S3) using time‐lapse confocal microscopy of the microtubule cytoskeleton in U2OS cells. For this purpose, we developed an image analysis routine that corrects for sample movement and fluctuations in irradiation intensity (see Figure S3 and the Methods section in the Supporting Information). Using various irradiation intensities (see the Methods section of the Supporting Information), we found that the covalent label shows moderate to high photobleaching after 100 imaging frames, whereas the fluorescence intensity was largely maintained for the dynamic label (Figure 1 B,C and Supporting Information, Figure S4). While the fluorescence intensity in samples with covalent labels dropped close to background at the highest irradiation intensity, it only showed a minor decrease in samples with dynamic labels (Figure 1 C Supporting Information, Figure S4).

Next, we compared the fluorescence intensity over time for both covalent and dynamic labeling in STED microscopy, using the same fluorophore Abberior STAR 635P (Figure 2 A). For covalent labels, we used a commercial antibody carrying multiple fluorophores and optimized for STED imaging. We found that under our experimental conditions and after 50 rounds of imaging, no fluorescence intensity in the STED images was detectable anymore. In contrast, the fluorescence intensity in samples with dynamic labels maintained approximately half of the initial intensity (Figure 2 A,B). Removal of oxygen through the addition of an oxygen‐scavenger system did not influence the intensity–time trace in the dynamic labeling approach (Supporting Information, Figure S5). We next compared the spatial resolution by measuring the microtubule diameter (FWHM) (Figure 2 C) and by applying Fourier ring correlation (FRC).^15^, ^16^, ^17^ While there was no difference in resolution for microtubule diameters, FRC indicated a slightly better resolution for the covalent label (75.3±1.1 nm) than for the dynamic label (89.7±0.6 nm) (Supporting Information, Figure S6). This difference could be explained by the higher noise sensitivity of FRC compared to FWHM analysis^17^ (Supporting Information, Figure S5 A). We extended the concept of dynamic labels to multicolor STED imaging by labeling two targets (mitochondria and microtubules) in the same cell using immunofluorescence. The secondary antibodies were labeled with different DNA docking strands (P5 and P1, see the Methods section in the Supporting Information). STED images were recorded with both complementary imager strands (labeled with Abberrior STAR 635P and Alexa Fluor 594, respectively) present in the imaging buffer, allowing for parallel recording of both spectral channels and using the same depletion laser of 775 nm (Figure 2 D and Supporting Information, Figure S7). The constant and rapid exchange of fluorophore labels bypasses photobleaching during the imaging process, which is beneficial for the acquisition of image stacks of mammalian cells (Supporting Information, Figure S8).

**Figure 2:**
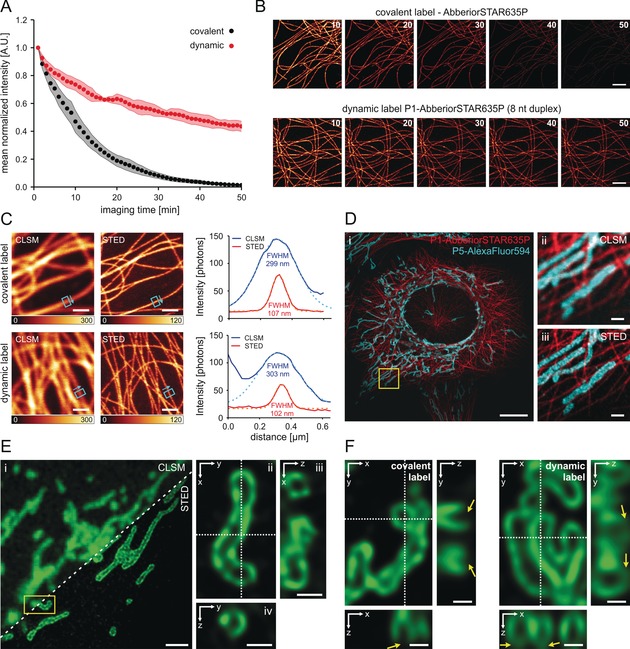
STED imaging using the transient binding of fluorophore‐labeled oligonucleotide probes. A) Intensity over time recorded on a STED microscope for the fluorophore AbberiorSTAR635P either as a covalent label (fluorophore‐labeled secondary antibody) or as dynamic label (DNA‐labeled secondary antibody; P1‐AbberiorSTAR635P, 500 nm). B) Representative time series of (A). Interval average images were generated from 10 frames, the number indicates the last frame of the interval. C) Comparison of CLSM and STED images acquired using covalent (upper row) and dynamic labels (lower row). Intensity bars indicate the photon counts for CLSM and STED images. Cyan rectangles and arrows indicate the position and direction of line profiles shown on the right. Gaussian fits to the intensity profiles (solid lines) are shown as dashed lines. D) Two‐color 2D‐STED imaging of microtubules (β‐tubulin, red) and mitochondria (TOM20, cyan) pseudo‐permanently labeled with 500 nm imager strands P1 and P5; i) overview STED image, ii) two‐color CLSM, and iii) two‐color STED images of the magnified region shown in (i) (yellow square). E) 3D‐STED imaging of mitochondria labeled for the detection of TOM20 (P5‐AlexaFluor594); i) comparison of CLSM and 3D‐STED images. The STED image shows the average image created from the entire deconvolved 3D‐STED *z*‐stack. ii) 120 nm xy‐slice (average from 4 *z*‐planes) of the single mitochondrium marked in (i) (yellow rectangle, 90° rotated). The orthogonal views in iii) xz and iv) yz reveal the hollow mitochondrial lumen. F) Comparison of 3D‐STED images using covalent (left) and dynamic labels (right). Yellow arrows indicate areas of out‐of‐focus photobleaching for the covalent label that causes loss of structural information, which does not occur using the dynamic label (scale bars are 10 μm (D part i), 2 μm (B, and E part i), 1 μm (C, and D parts ii and iii), and 0.5 μm (E parts iii and iv, and F)).

Next, we used dynamic labels for 3D‐STED microscopy of mitochondria labeled for the detection of TOM20 in U2OS cells (Figure 2 E). Dynamic labels allowed the recording of multiple images at subsequent axial positions with a 40 nm step size (see the Methods section of the Supporting Information), which provided STED images with near‐isotropic spatial resolution across the 3D volume. Compared to covalent labels, dynamic labels efficiently bypass out‐of‐focus photobleaching that especially compromises volumetric 3D‐STED microscopy with a high axial sampling rate (Figure 2 F).

In summary, we present a simple and powerful experimental protocol for STED microscopy in cells that bypasses photobleaching using DNA‐based, target‐specific, exchangeable fluorophore labels that transiently bind to their target. This concept can be extended by implementing DNA‐based labels, such as oligonucleotides conjugated to target‐specific aptamers.^18^ Previous work demonstrated STED imaging of DNA‐based fluorophore labels that permanently bind to a target, and achieved multiplexing by washing with dehybridization buffers.^11^ Similar procedures could be combined with the present approach using weak‐binding DNA labels, and further increase the multiplexing capability. Further development of this concept could include genetically expressed protein tags,^7^, ^19^, ^20^, ^21^ in particular with fluorophore labels engineered as weak binders and ultimately inside live cells.^7^ Adapting imaging parameters, such as integration time, line/frame averaging and pausing, allows the use of labels with very different exchange kinetics, hereby increasing the flexibility of the method.

## Conflict of interest

The authors declare no conflict of interest.

## Supporting information

As a service to our authors and readers, this journal provides supporting information supplied by the authors. Such materials are peer reviewed and may be re‐organized for online delivery, but are not copy‐edited or typeset. Technical support issues arising from supporting information (other than missing files) should be addressed to the authors.

Supplementary

Supplementary

Supplementary
