# Supplementary material for: Protein‐Specific, Multicolor and 3D STED Imaging in Cells with DNA‐Labeled Antibodies
Source: Angew Chem Int Ed Engl. 2019 Nov 7;58(52):18835–8. doi: 10.1002/anie.201910115 (PMC6972974; doi:10.1002/anie.201910115)
Supplement: Supplementary file 1 — Supplementary [file ANIE-58-18835-s001.pdf]

Angewandte Chemie
Eine Zeitschrift der Gesellschaft Deutscher Chemiker

Supporting Information

Protein-Specific, Multicolor and 3D STED Imaging in Cells with DNA-Labeled Antibodies

Christoph Spahn, Florian Hurter, Mathilda Glaesmann, Christos Karathanasis, Marko Lampe,* and Mike Heilemann**

anie_201910115_sm_miscellaneous_information.pdf
anie_201910115_sm_Video_1.mov
anie_201910115_sm_Video_2.mov

Supporting Information

Table of Contents

Experimental Methods 2Supplementary
Tables 8Supplementary
Figures 9Supplementary
Videos 16Reference
s 16

Experimental Methods

Cell Culture

U2OS cells were seeded on fibronectin-coated (Sigma Aldrich, Germany, 15 µg/ml fibronectin for 30 min) 8-well chamber slides (Sarstedt, Germany, 2 x 10^4 cells/well) and incubated at 37°C and 5% CO_2 in DMEM containing 4.5 g/l glucose, 10% FBS and 1% GlutaMAX (all purchased from Gibco, Thermo Fisher, USA). After 24 h, cells were chemically fixed using optimized protocols either for conservation of the microtubule cytoskeleton or conservation of microtubules and mitochondria. Single colonies of *E. coli* MG1655 WT cells (CGSC #6300) were picked from plate and grown overnight in LB Miller (Carl Roth, Germany) at 37°C. Cells were diluted 1:500 in 5 ml LB Miller and grown at 30°C to exponential phase while shaking (230 rpm).

Sample preparation

Cells were labeled with primary antibodies against tubulin (mouse anti-β-tubulin, #32-2600, Thermo Fisher, USA) and mitochondria (rabbit anti-TOM20, #sc-11415, Santa Cruz, USA). For secondary antibody labeling, we either used DNA-labeled antibodies (AffiniPure goat-anti-mouse #115-005-003 or AffiniPure donkey-anti-rabbit #711-005-152, both Jackson ImmunoResearch, USA), or commercial antibodies labeled with fluorophores (goat anti-mouse-Abberior STAR 635, Abberior, Germany and donkey-anti-rabbit-Alexa Fluor 594, #A32754, Thermo Fisher, USA).

<u>Fixation and labeling procedure for microtubules</u>

For labeling of microtubules, the cytosol of U2OS cells was extracted twice for 30-60 s using 37°C prewarmed microtubule stabilizing buffer (MTSB) (80 mM PIPES pH 6.8, 1 mM $MgCl_2$, 5 mM EGTA, 0.5% TX-100, Sigma Aldrich, Germany and Thermo Fisher, USA) (protocol published by Cramer and Desai [1]), followed by chemical fixation using MTSB + 0.5% GA (Electron Microscopy Sciences, USA) for 10 min. Cells were washed thrice with PBS and quenched for 7 min using 0.2% $NaBH_4$ (Sigma Aldrich, Germany) in PBS (Gibco, Thermo Fisher, USA). Cells were rinsed twice with PBS and subsequently washed twice with PBS + 1% BSA for 5 min. Microtubules were labeled using monoclonal mouse anti-β-tubulin antibody (see above) diluted to 1 µg/ml (CLSM experiments) or 5 µg/ml (STED experiments) in PBS containing 3% IgG-free BSA (Carl Roth, Germany). After 1 h incubation at RT, samples were washed thrice using PBS and custom-labeled donkey-anti-mouse secondary antibody (carrying P1 docking strand, 50 µg/ml in PBS + 3% IgG-free BSA) was added for 1 h . Samples were rinsed twice with PBS, washed twice for 5 min using PBS + 1% BSA and washed further 5 min with PBS. Finally, samples were post-fixed using 3% MeOH-free FA (Thermo Fisher, USA) and 0.2% GA in PBS (10 min @ RT) and washed thrice with PBS.

<u>Fixation and labeling procedure for microtubules and mitochondria</u>

Cells were fixed for 20 min at room temperature using 3% FA and 0.2% GA in PHEM buffer [2]. Afterwards, cells were washed thrice with PBS, quenched 7 min using 0.2% $NaBH_4$ in PBS, washed thrice with PBS and subsequently permeabilized for 20 min using PBS containing 3% IgG-free BSA and 0.25% TX-100. 5 µg/ml mouse anti-β-tubulin and rabbit anti-TOM20 primary antibodies (diluted in PBS + 3% BSA + 0.25% TX-100) were added simultaneously for 1 h. After washing with PBS/PBS + 1% BSA, custom-labeled secondary antibodies (P1-goat-anti-mouse; P5-donkey-anti-rabbit, see **Supplementary Table 1** for sequences) or a commercial secondary antibody (donkey-anti-rabbit-Alexa Fluor 594) were added for 1 h, followed by washing and post-fixing procedure described above.

Fixation and labeling procedure of bacterial cells

E. coli MG1655 cells were grown to OD_{600} ~ 0.5 (mass doubling time 36.8 ± 1.9 min) and fixed using 2% FA and 0.1% GA in $NaPO_4$ buffer for 15 min [4]. Cells were washed with PBS, incubated 30 min with PBS + 50 mM NH_4Cl and immobilized on KOH-cleaned (3M, 1h) and poly-L-lysine-coated (0.01% for 15 min) 8-well chamber slides. After immobilization, cells were blocked for 20 min at room temperature using PBS + 3% IgG-free BSA. 10 µg/ml polyclonal rabbit anti-*E. coli* antibody (BIO-RAD, #4329-4906) diluted in PBS + 1% BSA were added to each chamber. After 1 h incubation at room temperature, chambers were washed thrice with PBS and custom-labeled secondary antibodies (25 µg/ml P1-donkey-anti-rabbit or P5-donkey-anti-rabbit) diluted in PBS + 1% BSA were added for 1 h (RT). Cells were washed thrice with PBS, twice with PBS + 1% BSA, once again with PBS and post-fixed for 10 min at room temperature using 2% MeOH-free FA in PBS. Finally, chambers were washed once with PBS and excess formaldehyde was quenched for 20 min using PBS + 50 mM NH_4Cl.

Labeling of antibodies with DNA docking strands

DNA-labeled secondary antibodies were prepared according to a published protocol [3]. In brief, 30 µL of 1 mM thiolated DNA (P1 docking strand: HS-TTA TAC ATC TA, P5 docking strand: HS-TTT CAA TGT AT; Eurofins Scientific, Luxembourg) in PBS were added to 70 µl of 250 mM dithiothreitol (DTT; Thermo Fisher, USA) and 1.5 mM ETDA in 0.5x PBS at pH 7.2 (Sigma Aldrich, Germany), stirred for 2h at room temperature, and purified with a Nap-5 column (GE Healthcare, USA) pre-equilibrated with ddH_2O. A solution of the secondary antibody was mixed with the crosslinker maleimide-PEG2-succinimidyl ester (M = 425.39 g/mol, linker 17.6 Å, Sigma-Aldrich, Germany, #746223) in a 10:1 ratio, incubated for 90 min at 4°C, and desalted and washed using a Zeba desalting column (Zeba Spin Desalting Column, 7k MWCO, Thermo Fisher, USA). The antibody solution and DNA solution were mixed in a 1:10 ratio and incubated overnight at 4°C while shaking. Excess DNA was removed with a spin filter (100 kD, Amicon; Sigma Aldrich, Germany), washing several times with PBS.

Fluorophore-labeled imager strands (P1-10nt, TAG ATG TAT-STAR635P (9 bp duplex), P1-8nt, AG ATG TAT-STAR635P (8 bp duplex), P5-10nt C ATA CAT TGA-Alexa Fluor 594 (9 bp)) were ordered from IBA Lifesciences (Germany).

Confocal microscopy

Time series were recorded on a Zeiss LSM710 bearing a Plan-Apo 63x oil objective (DIC M27, 1.4 NA) (Zeiss, Germany). An internal 633 nm diode laser was used for excitation at varying laser intensities, ranging between 15.5 µW and 156 µW (10% and 90% of maximal laser power, respectively). A heating module consisting of a TempModule S (Zeiss, Germany) and a heating insert P (PeCon GmbH, Germany) were used to maintain a temperature of 25°C, which is crucial to maintain constant hybridization/dissociation rates of the docking/imager DNA oligo pairs and to reduce sample drift. The microscope was controlled using Zeiss ZEN 2008 software (v. 5,0,0,267).

Imaging was performed in hybridization buffer consisting of PBS (without Ca^{2+} and Mg^{2+}) + 500 mM NaCl pH 8.2 (adjusted using NaOH). 100 imaging frames were recorded for each intensity time trace, setting a pixel size of 87.5 nm (22.4 x 22.4 µm region of interest), a pixel dwell time of 12.61 µs/px, 2x line averaging and a pinhole size of 1 airy unit. The master gain was set to a value of 550 - 800, depending on the laser intensity used, in order to achieve a good contrast while preventing signal saturation. 5 (covalent label) - 15 (dynamic label) time course measurements were acquired and analyzed for each laser intensity.

Confocal measurements contributing to Figure S2 were recorded on TCS SP8 confocal microscope (Leica Microsystems, Germany). Exact parameters for each measurement are listed in **Supplementary Table 2.**

STED Microscopy

STED images were acquired with an inverted TCS SP8 3X microscope (Leica Microsystems, Germany) equipped with a 100x/1.4 NA oil immersion objective (Leica HC PL APO CS2 – STED White) or a 86x/1.2 NA water immersion objective (Leica HC PL APO CS2 – STED White), operated by the Leica LAS X software (version 3.1.5.16308). Fluorophores were excited with 594 nm (AlexaFluor594) or 633 nm (AbberiorSTAR635P) laser light derived from an 80 MHz pulsed White Light Laser (Leica Microsystems, Germany). Stimulated emission was performed with a 775 nm pulsed laser (Leica Microsystems, Germany). Fluorescence emission was collected with Hybrid Detectors (HyD, Leica Microsystems, Germany) using a gate of 0.3-6 ns with respect to the excitation pulse (see **Supplementary Table 2** for detection windows). Dual color images of AlexaFluor594 and AbberiorSTAR635P was performed in line sequential mode. Pixel size was set to 20 - 30 nm for 2D-STED microscopy and ~40 nm for 3D-STED microscopy if not stated otherwise. The pinhole was set to 0.7-1.0 AU for 2D-STED microscopy and closed down to minimum 0.53 AU in 3D-STED. Images were typically recorded in photon counting mode or standard mode using 2-16x line accumulation or line averaging, respectively. Dwell times were kept in the range of 0.6-1.525 µs. The microscope was equipped with an incubation chamber (constructed in-house at EMBL Heidelberg) and a temperature of

22.5 ± 0.2 °C was ensured by constant cooling to minimize sample drift and optimize optical performance. Exact parameters for each measurement are listed in **Supplementary Table 2**.

For STED imaging, a final concentration of 500 nM of fluorophore-labeled imager strands (see Supplementary Table 1), diluted in the hybridization buffer as used for CLSM imaging was added to the immunostained samples and three time course measurements were recorded for both the covalent and dynamic labeling approach (1 min/frame, 50 frames). For Figure S5B, an oxygen scavenger system consisting of 10 nM protocatechuate- 3,4- dioxygenase (PCD, #03930590, Sigma Aldrich, Germany) and 2.5 mM protocatechuic acid (PCA, # P8279, Sigma Aldrich, Germany) was added to the imaging buffer.

Image analysis

Analysis of intensity time traces

Image stacks were exported in TIFF format and further processed using the open-source image analysis package Fiji (v1.51w). Image stacks of fixed cells (z-stacks or multi-frame measurements) were aligned using the Fiji plugin "Stackreg" (translation). Intensity time traces were extracted using a custom-written Fiji macro. In short, an average image was calculated from stabilized time series using the plugin "Z project". The resulting image was manually thresholded and the resulting binary image was segmented using the plugin "Watershed" to prevent inclusion of label-free regions in the analysis. Further, 8 pixels at the image borders were excluded, as they contain artifactual intensity values resulting from the alignment procedure. Resulting ROIs were combined in the "RoiManager" and mean pixel values were extracted for each frame in the original time series. The binary image was inverted in order to obtain the background ROI. We found that the background introduced by freely diffusing imager strands is a reliable indicator for fluctuations in laser intensity. The average background pixel value was determined for each imaging frame and both signal and background values were automatically saved as text files. The obtained raw data was then further processed using OriginPro 2018 (OriginLabs). Intensity traces of both signal and background were normalized to the first frame. Signal intensity traces were then corrected by division with the background intensity trace. The resulting corrected intensity traces were averaged and plotted with the respective standard deviation. Time series of the covalent label were processed similarly, except that no background correction was applied due to the lack of an indicator for laser intensity fluctuations.

Image deconvolution

Image deconvolution was carried out using Huygens Professional version 16.10.1p2 (Scientific Volume Imaging).

Determination of microtubule diameters

Diameters of microtubules were determined by measuring the full-width-at-half-maximum (FWHM) perpendicular to straight filaments using the Fiji plugin "Plot Profile" and a line width of 400 nm. FWHM values were extracted using a Gaussian fit function in OriginPro 2018.

Determination of signal-to-background and signal-to-noise ratios

The mean pixel values for signal and background were determined as described above and shown in Figure S3. For CLSM images, background intensity was determined ~ 2 - 3 µm above the glass surface to avoid contributions by antibodies/labels unspecifically bound to the glass surface. Noise was determined as the standard deviation of the mean background pixel value in each image.

Fourier ring correlation analysis

Fourier ring correlation (FRC) was performed using the Fiji plugin written by Olivier Burri and Alex Herbert (https://imagej.net/Fourier_Ring_Correlation_Plugin#Installation). Image pairs were created from the first 10 frames of STED bleaching time series contributing to Figure 1A (image 1: frames 1, 3, 5, 7, 9; image 2: frames 2, 4, 6, 8, 10). A correlation cutoff of 1/7 was used to determine the spatial resolution for each image pair. 3 measurements were analyzed for both the covalent and dynamic label.

DNA-PAINT imaging

DNA-PAINT imaging of the *E. coli* K12 envelope was performed on a commercial N-STORM setup (Nikon, Japan). This setup comprises a Nikon Eclipse Ti body, a motorized XY-stage (TI-S-ER), a perfect focus system (TI-PFS) and an Apo TIRF 100x oil objective (NA 1.49) (all Nikon, Japan). As excitation source, a multi-laser combiner (MLC400, Agilent Technologies, USA) is fiber-coupled into the microscope and fluorescence emission is detected on an Andor iXon Ultra EMCCD camera (DU-8974-CS0#BV, Andor Technology, Northern Ireland). Lasers were controlled using the NIS Elements (version 4.30.02) and camera using the µManager software (version 1.4.14). For imaging of P5-AlexaFluor594, a zt561rdcxt beamsplitter (AHF Analysentechnik, #F43-T07) and ET 605/70 bandpass emission filter (AHF Analysentechnik, Germany, #F47-700) were used, while P1-AbberiorSTAR635P was recorded using a T660 LPXR beamsplitter (AHF Analysentechnik, Germany, #F48-660) and ET 700/75 bandpass emission filter (AHF Analysentechnik, Germany, #F47-700). 150 - 300 pM imager strand in hybridization buffer were added to the samples and 20k frames were recorded at 8 Hz frame rate (camera settings: preamplifier gain 1, EM-gain 200, 17 MHz readout, frame transfer mode, 158.3 nm pixel size). AlexaFluor594 was excited using 561 nm laser light at 0.45 kW/cm^2 and AbberiorSTAR635P using 0.3 kW/cm^2.

DNA-PAINT analysis

Single-molecule localization data was analyzed using Picasso v. 0.28 [3]. Hybridization events were localized using the LQ Gaussian fitting algorithm with a box size of 7 px and a min. net. gradient of 30k. Data were drift corrected using RCC (500-1000 frames window size) and subsequent frame localizations within 0.5 px (~80 nm) were grouped allowing a dark time of 2 frames. Localizations were

filtered according to their PSF width in x and y direction ($95 < \rho_{PSF} < 205$ nm) in order to reduce out-of-focus signal. The number of tracked frames was taken as a measure for the emitter ON time (binding time). At least 60,000 single-molecule spots recorded in at least 2 measurements were analyzed to determine the binding time for the imager strands. Binding times were approximated by fitting the distribution with a mono-exponential decay function omitting the first two time intervals (OriginPro 2018).

Supplementary Note 1

Relative binding times of the imager strands used in this study were determined using *E. coli* MG1655 WT cells labeled for surface exposed K12 epitopes. We chose this target as *E. coli* cells exhibit a diameter of ~ 1 µm, resulting in good spatial separation of individual hybridization events when imaging the bacterial mid plane. This is superior to DNA-PAINT imaging of e.g. TOM20, as mitochondria show varying diameters, invaginations and constriction sites, thus increasing the fraction of overlapping single-molecule events. Furthermore, filtering of signals according to their PSF width successfully removes out-of-focus events and background localizations which is crucial for robust determination of imager strand binding times. We did not choose DNA-origami for this experiment, as different imaging buffers are used for their imaging (e.g. tris-buffer containing magnesium for DNA-origami stabilization) and we aimed to use the hybridization buffers applied in CLSM and STED imaging.

Supplementary Tables

Supplementary Table 1

Supplementary Table 1: Docking and imager strands used in this study. The complementary sequence of the imager strand is shown bold and colored

ssDNA oligo	Sequence
P1 docking strand	3’ - ATC TAC ATA TT - 5’ - antibody
P1 imager strand (9 nt duplex)	5’ - C **TAG ATG TAT** - 3‘ - AbberiorSTAR635P
P1 imager strand (8 nt duplex)	5’ - **AG ATG TAT** - 3‘- AbberiorSTAR635P
P5 docking strand	3’ - TAT GTA ACT TT - 5’ - antibody
P5 imager strand (9nt duplex)	5’ - C **ATA CAT TGA** - 3‘ - AlexaFluor594

Supplementary Table 2

Supplementary Table 2 is provided as separate .xlsx file and comprises a detailed overview on the imaging conditions for each experiment.

Supplementary Figures

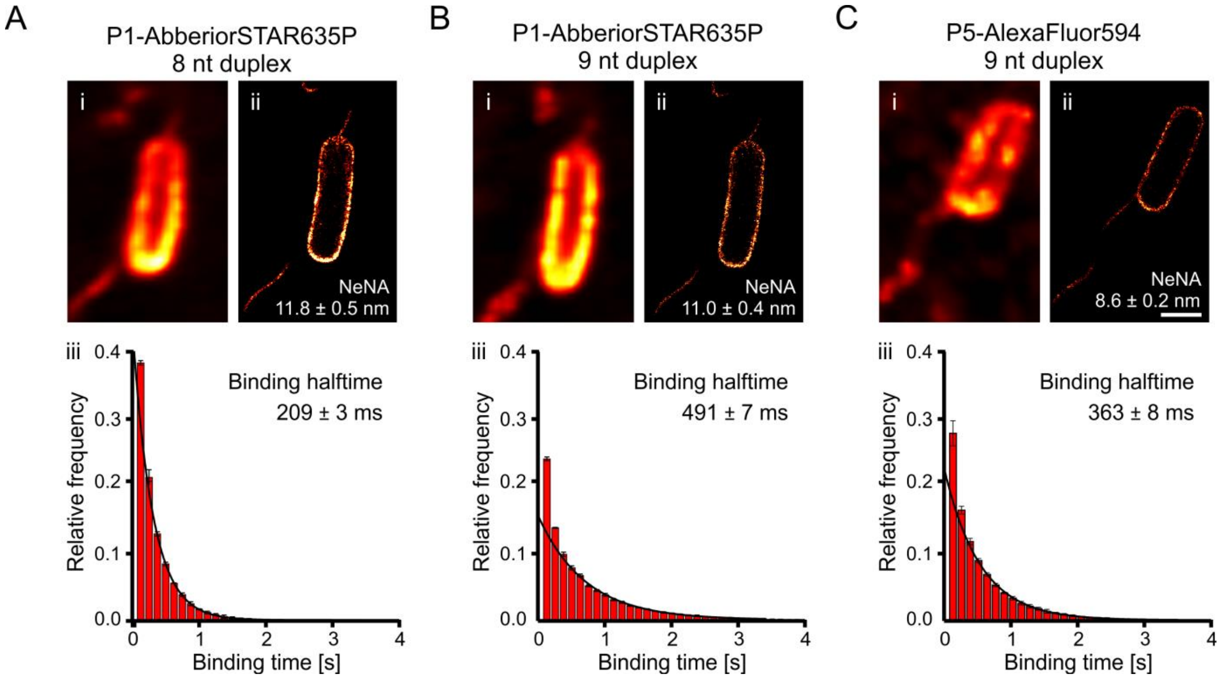

Figure S1: Determination of imager strand binding times using single-molecule DNA-PAINT imaging. The K12 envelope of *E.coli* MG1655 cells were visualized with DNA-PAINT using the 8 nt [A] and 9 nt [B] duplex forming P1-AbberiorSTAR635P imager strand, as well as the 9 nt duplex forming P5-AlexaFluor594 imager strand [C]. Shown are representative diffraction limited images (i) (standard deviation images of DNA-PAINT movies) and the respective super-resolved DNA-PAINT images (ii). Low irradiation intensities (0.3 and 0.45 kW/cm² for AlexaFluor594 and AbberiorSTAR635P, respectively) were used to minimize photobleaching of hybridized imager strands while still bound to the docking strand. Pronounced bleaching of bound imager strands would lead to an artificial shortening of the binding time. Still, the experimental localization precision (NeNA value [5]) were in the range of 8-12 nm. For each imager strand, binding times were extracted (> 60,000 events per strand). Fitting the binding time distribution with a mono-exponential decay (iii) provided binding halftimes of 209 ± 3 ms (P1-AbberiorSTAR635P, 8 nt duplex), 491 ± 7 ms (P1-AbberiorSTAR635P, 9 nt duplex) and 363 ± 8 ms (P5-AlexaFluor594, 9 nt duplex). Scale bar is 1 µm.

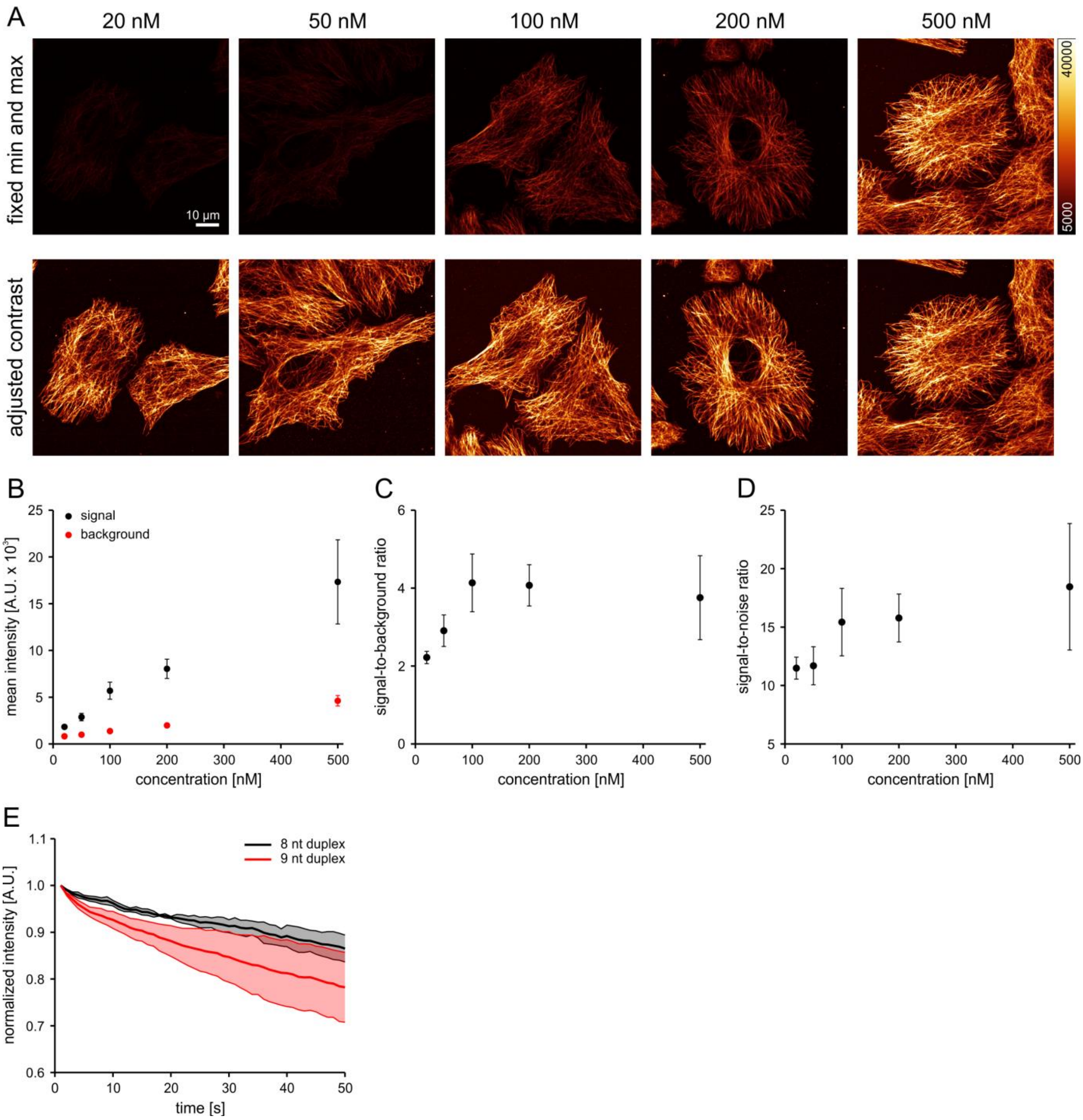

Figure S2: Concentration and duplex length optimization of dynamically binding DNA labels. [A] Signal-to-noise ratio was optimized by titration imager strand concentrations ranging from 20 nM to 500 nM of the 8 nt duplex forming DNA-PAINT probe (P1-AbberiorSTAR635P) to anti-tubulin labeled samples in PBS (pH 8.2) + 500 mM NaCl. Representative images recorded with 8x frame accumulation on a CLSM are shown. Images are displayed both using absolute intensities for comparison (upper row) and contrast-adjusted (lower row) to pronounce label quality. [B] Dependence of signal (black dots) and background (red dots) on label concentration. Mean signal and background values were determined as described in the methods section. [C] Signal-to-background and [D] signal-to-noise ratio in dependence of label concentration. [E] Optimization of imager strand length by minimizing signal-decrease over time. Signal over time for 8 (black lines and shaded areas) and 9 nt (red lines and shaded areas) duplex forming labels, recorded at 1 Hz frame rate (CLSM). Three measurements were analyzed for each condition. All plots show mean values (dots and solid lines) and the respective standard deviations (error bars and shaded areas).

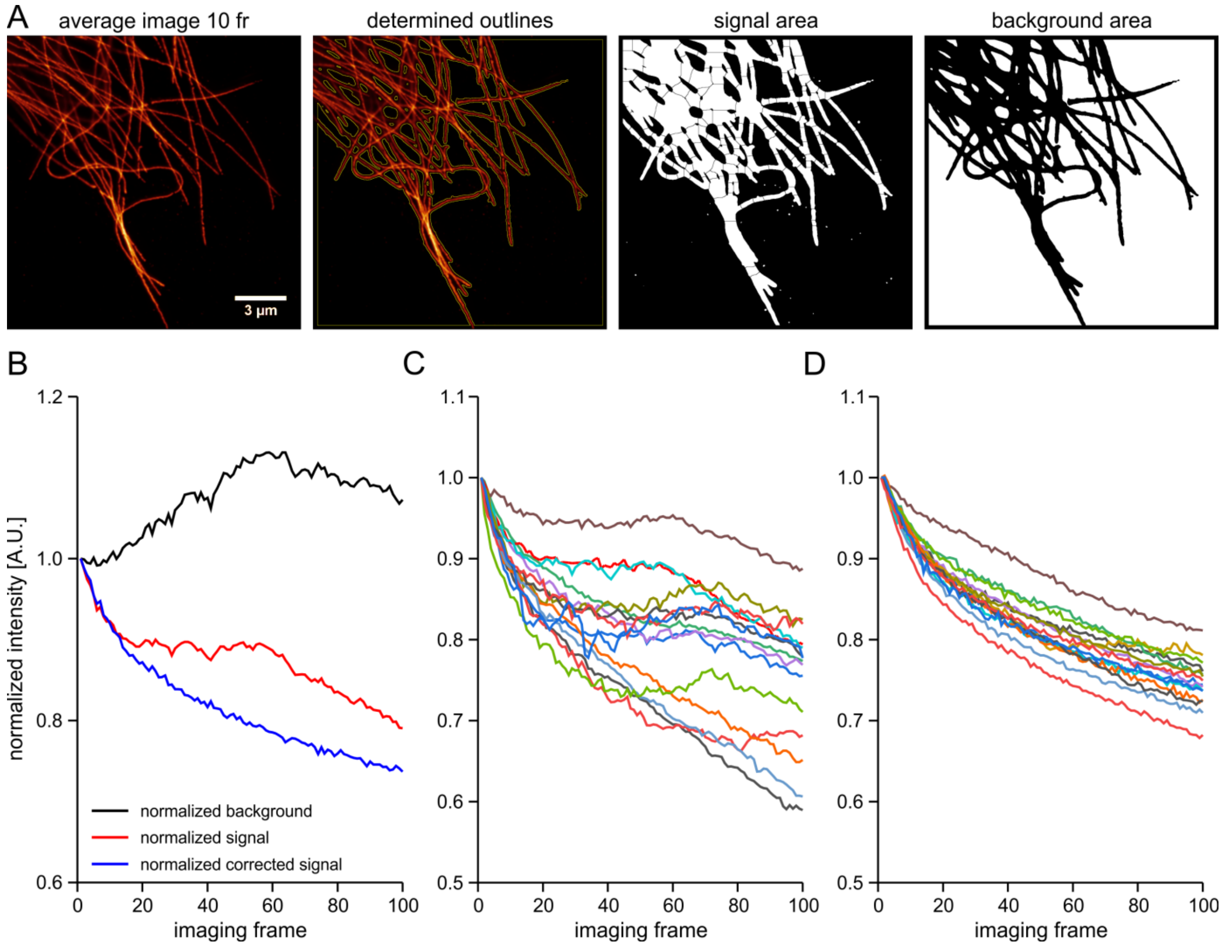

Figure S3: Extracting intensity time traces from multi-frame image stacks. [A] Extraction of signal and background intensity from time series. An average image was calculated from the first 10 frames and was used to determine the outlines of the labeled structure. The thresholded image was segmented and the resulting regions of interest of both signal and background area were transferred to the time series to extract intensity values for each imaging frame. Edge pixels were excluded to prevent artifacts that might be introduced by sample drift correction. [B] An example of intensity time traces for uncorrected signal intensity (red line), background intensity (black line) and the background-intensity corrected signal, deliberately showing an example with strong intensity fluctuations. The correction procedure compensated for fluctuations in laser power (see methods). [C] Uncorrected intensity time traces of confocal image stack recorded at 90% relative laser power (156 µW). [D] Corrected intensity time traces shown in [C].

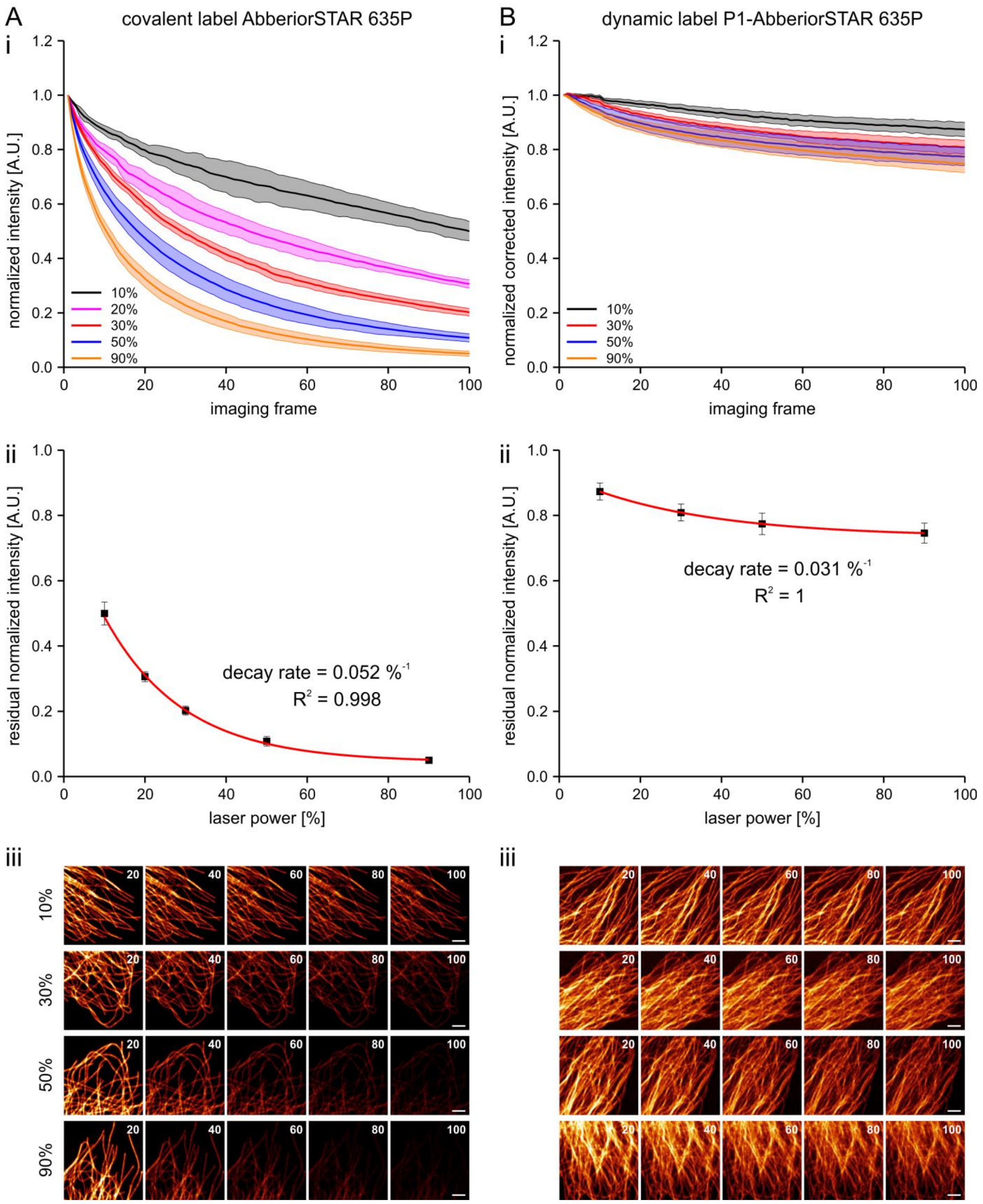

Figure S4: Influence of laser power on intensity over time for covalent [A] and dynamic labeling [B] measured from U2OS cells labeled for microtubules. [i] Normalized intensity time traces, showing a reduced sensitivity to photobleaching for dynamic labeling. Note that due to the lack of diffusing imager strands for the covalent label, fluctuations in laser intensities could not be quantified and hence not be corrected. This leads to increased standard deviations compared to the corrected time traces of the dynamic label. Curves show the mean values of 5 - 15 intensity time traces with respective standard deviations shown as colored areas. [ii] Influence of the excitation laser power on the residual intensity after 100 imaging frames (last imaging frame in [i]). Both labeling strategies display a mono-exponential intensity decay (red line), with the dynamic label decaying less than the covalent label and showing a much higher residual intensity (relative to the first frame) after 100 consecutive imaging frames. [iii] Representative time series for covalently (left panel) and dynamically (right panel) labeled microtubules recorded at varying laser intensities. Shown are the interval average images created from 20 successive imaging frames with numbers indicating the last imaging frame of this interval. Scale bars are 3 µm.

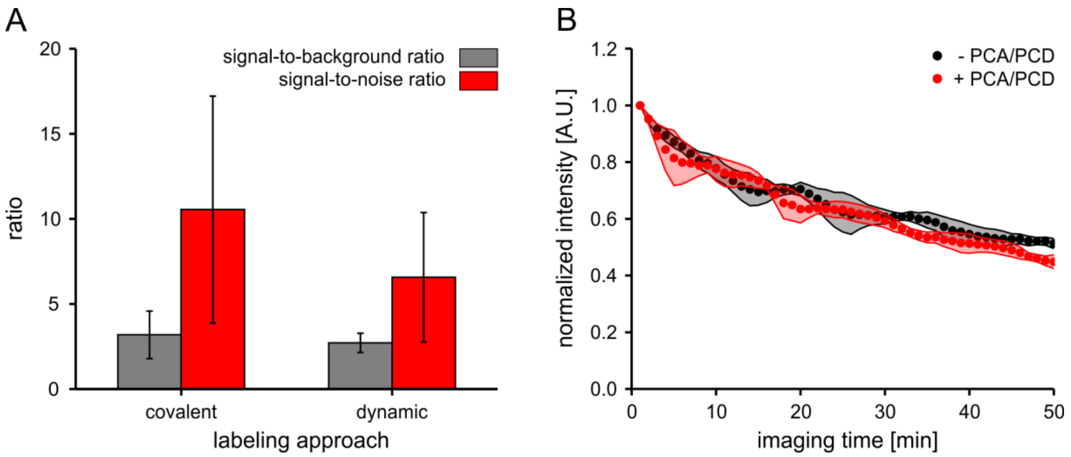

Figure S5: Comparison of signal-to–background and signal-to-noise ratios in STED microscopy using covalent and dynamic labels and evaluation of the oxygen scavenger system PCA/PCD. [A] Signal-to-noise and signal-to-background ratios of STED measurements using the covalent or dynamic labeling approach (an exemplary ROI is shown in Figure 1C). Signal to background (3.2 ± 1.4 vs. 2.7 ± 0.6) and signal to noise ratios (10.6 ± 6.7 vs. 6.6 ± 3.8) were slightly higher for the covalent label compared to the dynamic label. Note that this could be explained by an increased degree of labeling for secondary antibodies carrying AbberiorSTAR635P dyes. [B] Signal over time in STED time series using 500 nM P1-AbberiorSTAR635P (8 nt duplex) without (black dots and shaded areas) and with (red dots and shaded areas) the PCA/PCD oxygen scavenger system. The removal of oxygen has no apparent effect on the intensity time trace. Three measurements were analyzed for each condition. Plots show mean values (bars and dots) and the respective standard deviations (error bars and shaded areas).

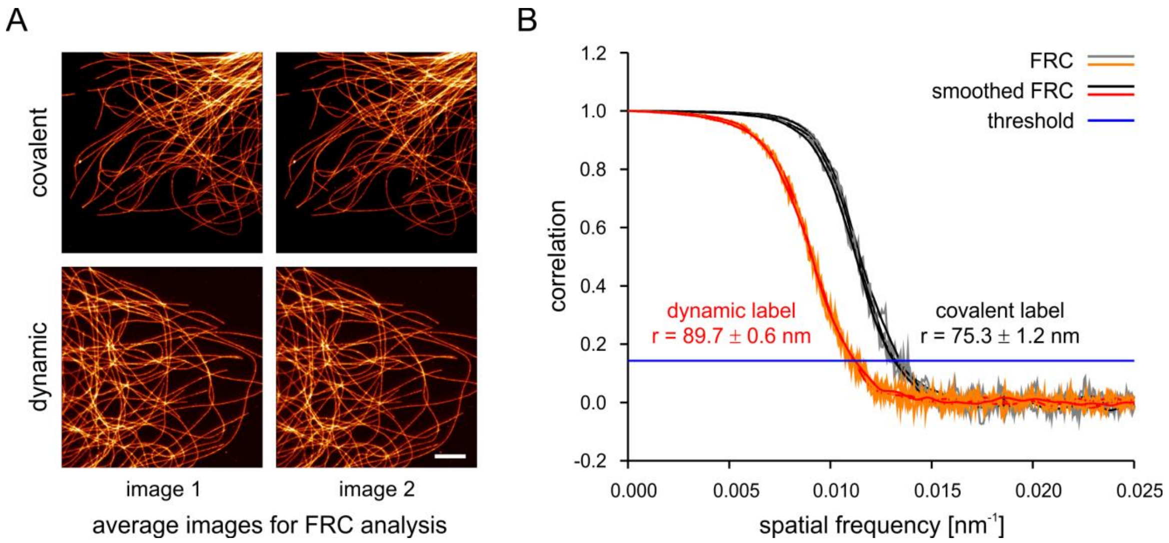

Figure S6: Fourier ring correlation analysis of covalent and dynamic labels. [A] Example image pairs for static and dynamic labeling of β-tubulin using AbberiorSTAR635P. [B] Resulting FRC plots from 3 independent measurements. Scale bar in [A] is 3 µm.

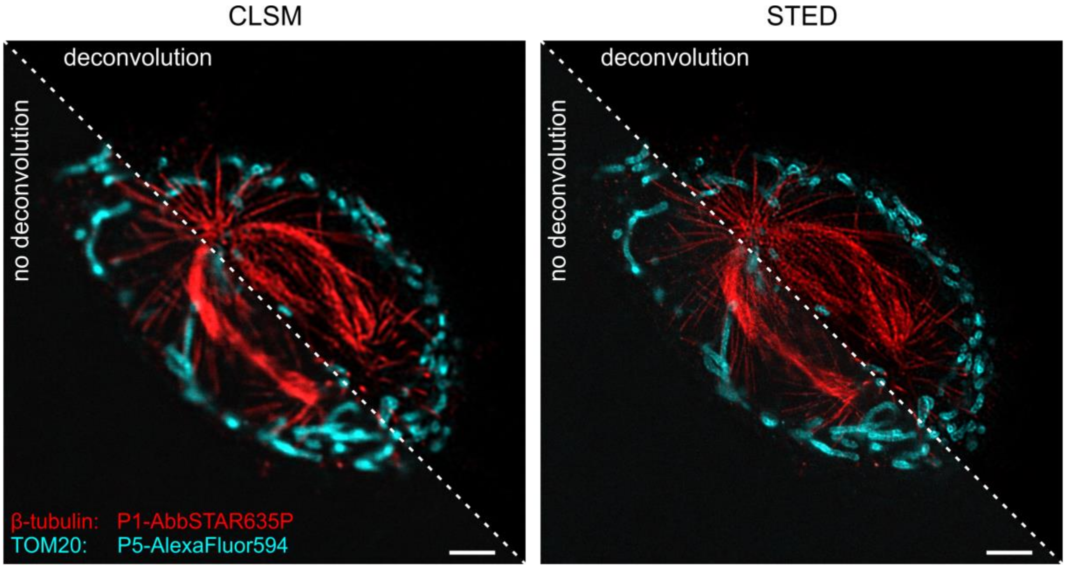

Figure S7: Dual-color 2D STED of a single plane of a mitotic cell recorded approximately 5 µm above the glass surface. CLSM (left) and STED images (right) were recorded using 500 nM imager strands in hybridization buffer. Further details can be revealed by applying image deconvolution algorithms. Scale bars are 3 µm.

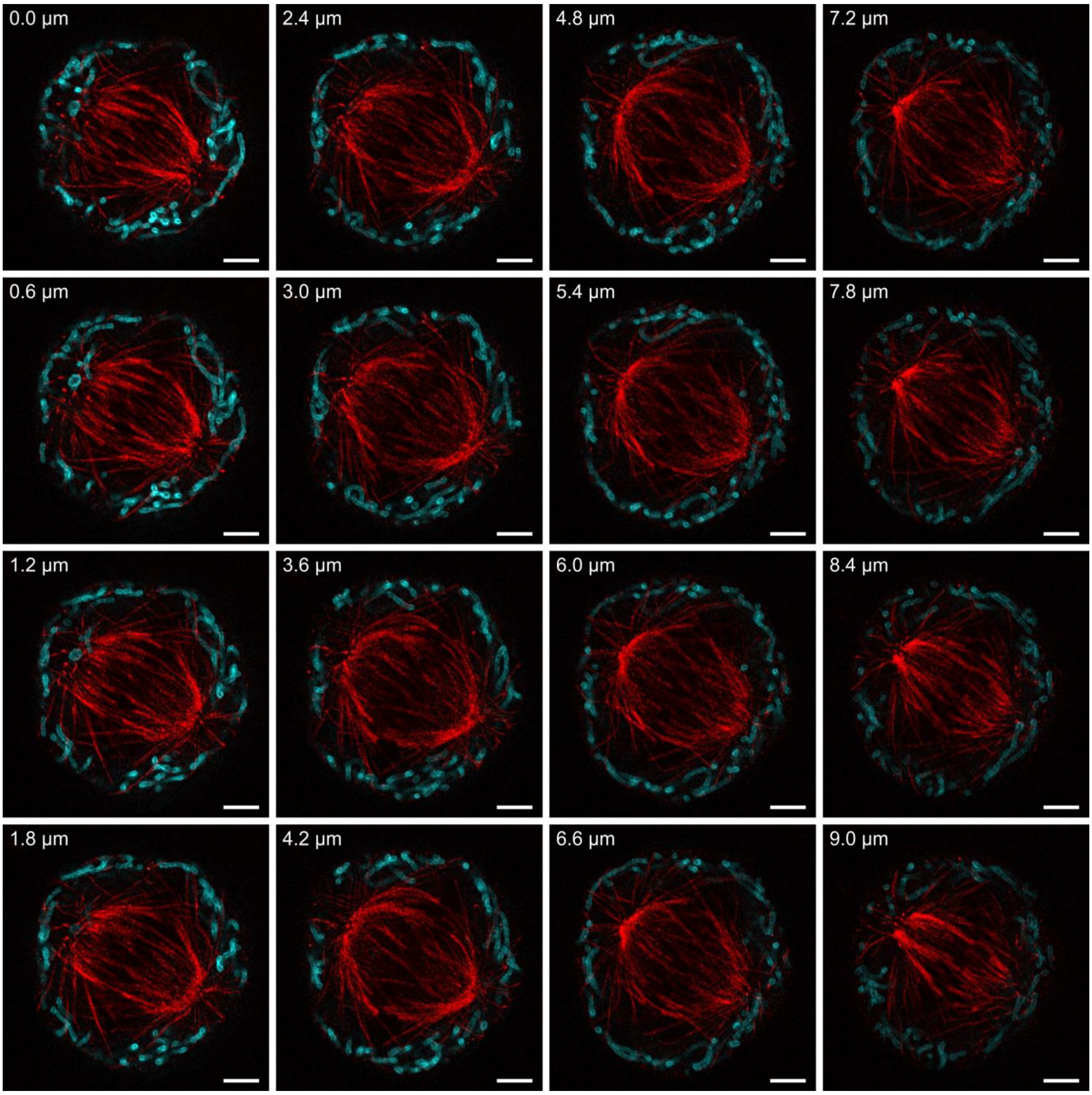

Figure S8: Dynamic labeling allows recording stacks of STED images along a large axial range. z-stack of a mitotic HeLa cell labeled for β-tubulin (red, 500 nM P1-AbberiorSTAR635P) and TOM20 (cyan, 500 nM P5-Alexa Fluor 594). Images represent average 2D-STED images of 2 planes. A comparison between the CLSM and STED images is shown in Supplementary Video 1. Scale bars are 3 µm.

Supplementary Videos

Supplementary_Video_1.mov

Title: Supplementary Video 1: Two-color STED of U2OS labeled for tubulin and TOM20 with transiently binding DNA-oligomers.

Legend: Dual-color CLSM (left panel) and STED z-stack (right panel) of a mitotic cell. Tubulin (red) was visualized using P1-AbbSTAR635P imager strand (8 nt duplex) and TOM20 (cyan) using P5-AlexaFluor594 (9 nt duplex). Two planes (600 nm in total) were averaged, numbers indicate the relative axial position. Scale bar is 3 µm.

Supplementary_Video_2.mov

Title: Supplementary Video 2: Volumetric rendering of a 3D STED image of a single mitochondrion.

Legend: TOM20 was visualized using P5-AlexaFluor594. Moderate deconvolution and smoothing was performed (see Supplementary Table 1).

References

[1] Cramer and Desai, "Fluorescence Procedures for the Actin and Tubulin Cytoskeleton in Fixed Cells", https://mitchison.hms.harvard.edu/files/mitchisonlab/files/

[2] Legant *et al.*, "High density three-dimensional localization microscopy across large volumes", *Nature Methods* 13(4), 359-365 (**2016**)

[3] Schnitzbauer *et al*, " Super-resolution microscopy with DNA-PAINT", *Nature Protocols* 12, 1198–1228 (**2017**)

[4] Spahn *et al.*, "A toolbox for multiplexed super-resolution imaging of the E. coli nucleoid and membrane using novel PAINT labels", *Scientific Reports* 8, 14768 (**2018**)

[5] Endesfelder *et al.*, "A simple method to estimate the average localization precision of a single-molecule localization microscopy experiment", *Histochem. Cell Biol.* 141(6), 629-38 (**2014**)
